# Induction of Non-Canonical Ferroptosis by Targeting Clusters Suppresses Glioblastoma

**DOI:** 10.3390/pharmaceutics16091205

**Published:** 2024-09-13

**Authors:** Kai Cao, Liyuan Xue, Kaidi Luo, Wendi Huo, Panpan Ruan, Dongfang Xia, Xiuxiu Yao, Wencong Zhao, Liang Gao, Xueyun Gao

**Affiliations:** Center of Excellence for Environmental Safety and Biological Effects, Department of Chemistry, College of Chemistry and Life Science, Beijing University of Technology, Beijing 100124, China

**Keywords:** gold clusters, glioblastoma, non-canonical ferroptosis, nrf2, heme oxygenase-1, anti-tumor T cell response

## Abstract

Glioblastoma multiforme (GBM) is the most aggressive brain tumor. There is a pressing need to develop novel treatment strategies due to the poor targeting effect of current therapeutics. Here, a gold cluster coated with optimized GBM-targeting peptide is engineered, namely NA. NA can efficiently target GBM both in vitro and in vivo. Interestingly, the uptake of NA significantly sensitizes GBM cells to ferroptosis, a form of programmed cell death that can bypass the tumor resistance to apoptosis. This effect is exerted through regulating the HO-1-dependent iron ion metabolism, which is the non-canonical pathway of ferroptosis. The combined treatment of a ferroptosis inducer and NA profoundly inhibited tumor growth in both the GBM spheroid model and a syngeneic mouse model with enhanced ferroptosis levels and excellent biosafety. Importantly, the infiltration of tumoricidal lymphocytes is also significantly increased within tumor. Therefore, NA presents a potential novel nanomaterial-based strategy for GBM treatment.

## 1. Introduction

Glioblastoma (GBM) is the most common and aggressive primary brain tumor in adults, accounting for approximately 15% of all intracranial tumors and contributing significantly to mortality associated with brain cancers [1]. This malignancy arises from glial cells and is characterized by rapid growth and a highly invasive nature, making it difficult to treat effectively. GBM occurs most frequently in the cerebral hemispheres and can metastasize to areas outside brain, such as the lungs, liver, and skeletal system [2]. The median survival time of patients with GBM is only 12 to 15 months despite aggressive treatment strategies, including surgery, radiation, and chemotherapy [3]. Based on the poor prognosis and limited efficacy of current therapies, there is a pressing need for the development of new, more effective treatment options for patients with glioblastoma.

Nanomaterials have gained significant attention in cancer treatment [4]. In the context of GBM treatment, research has predominantly focused on developing nanocarriers to cross the blood–brain barrier (BBB) [5]. However, the use of nanomedicines specifically designed for GBM remains relatively underexplored. Ultra-small gold clusters are a notable category of gold-based nanomaterials, characterized by tunable fluorescence, designable peptide coatings, excellent biocompatibility, and intrinsic biomedical activities, making them highly promising for biomedical applications [6]. The gold element within gold clusters has been demonstrated to affect various biological processes to treat cancer, while the coating peptide can be designed to target specific tumor types, thus enhancing the specificity and safety [7,8,9,10,11]. Additionally, several studies from ours and others have proven that gold clusters can efficiently penetrate the BBB) [12,13,14,15]. Therefore, GBM-targeting peptide-conjugated gold clusters possess great potential in the treatment of GBM, not only metastatic glioblastoma outside brain, but also in situ GBM.

Ferroptosis is a form of programmed cell death characterized by the accumulation of lipid peroxides and iron-dependent oxidative damage, distinct from apoptosis and other cell death mechanisms [16]. In GBM treatment, ferroptosis has emerged as a promising therapeutic avenue due to its unique mechanism of action, which can bypass the genetic and molecular alterations that often confer resistance to traditional apoptosis-inducing chemotherapeutics [17]. However, the induction of ferroptosis also poses risks, as this process can contribute to the pathology of diseases such as cardiovascular disorders [18]. Therefore, it is crucial to develop strategies that selectively target tumor cells while minimizing the off-target effects of ferroptosis inducers. This balance is vital to maximizing the therapeutic efficacy against GBM while minimizing potential systemic toxicity.

Gold-based nanomaterials have been shown to regulate reactive oxygen species (ROS), which is the main regulator of ferroptosis [19,20,21,22]. ROS during ferroptosis can be generated by either GPX4 reduction or the Fenton reaction of Fe^2+^, representing the canonical and non-canonical ferroptosis pathways, respectively [16]. We have shown that gold clusters can promote the expression of blood oxygenation enzyme-1 (heme oxygenase-1, Hmox1/HO-1), which is critical for Fe^2+^ metabolism [7]. These findings suggest the potential utility of gold clusters in the regulation of non-canonical ferroptosis.

In this study, we synthesized a GBM-targeting peptide-conjugated gold cluster (namely NA) with an excellent tumor-targeting ability. Compared with a non-targeting gold cluster, NA can increase the concentration of Fe^2+^ in GBM tumor cells by promoting the expression of HO-1, thereby promoting lipid peroxidation and ferroptosis through the non-canonical mechanism. More importantly, NA combined with a ferroptosis inducer could exert a significant anti-tumor effect in a subcutaneous tumor model by inducing ferroptosis and promoting anti-tumor T cell responses, with little cardiotoxicity. Combined with the BBB-crossing property of NA, our results provide theoretical support for the development of new nanomaterial-based strategies for the treatment of both metastatic and in situ GBMs.

## 2. Materials and Methods

### 2.1. Cell Lines

Mice cell line GL261, human malignant glioblastoma cells (U87MG), and human astrocytoma cells (U251) were obtained from the national experimental cell resource sharing platform (NICR, Beijing, China). GL261 cells were cultured in Dulbecco’s modified Eagle’s medium-H (DMEM-H) supplemented with 10% inactivated FBS, 100 U/mL penicillin, and 100 µg/mL streptomycin in 5% CO_2_ at 37 °C. U87MG and U251 cells were cultured in Minimum Essential Medium (MEM) supplemented with 10% inactivated FBS, 100 U/mL penicillin, and 100 µg/mL streptomycin in 5% CO_2_ at 37 °C.

### 2.2. Preparation of Gold Nanoclusters

GA was prepared according to our previous report [7]. The method used for the preparation of NA was one-step synthesis. Briefly, 10 mg Nar peptide (Hefei Synthbiological Engineering, Hefei, China) was dissolved in 7.69 mL of ultrapure water, and after complete dissolution, 61.4 μL HAuCl_4_ (25 mM) and 307.2 μL NaOH (0.5 M) were slowly added and rapidly stirred for 10 min. The mixture was then stirred for 7 h at 55 °C. The mixture was stored at room temperature in the dark for 12 h. The prepared samples were purified with an ultrafiltration tube with a molecular weight cutoff of 3 kDa to remove unreacted peptides and gold. Purified samples should be stored at 4 °C for later use. The concentration of nanoclusters was quantified using inductively coupled plasma mass spectrometry (ICP-MS, PerkinElmer, Shelton, MA, USA).

### 2.3. Characterization of Gold Nanoclusters

The fluorescence spectrum of NA was detected using a Shimadzu RF-5301 fluorescence spectrophotometer (Kyoto, Japan). UV-Vis absorption spectra were measured using a Shimadzu UV-1800 photo spectrometer (Kyoto, Japan). The particle size and potential of the gold nanocluster NA were determined using a Malvern laser particle analyzer (ZSE, Malvern, UK). High-resolution transmission electron microscopy (HRTEM, Tecnai F20, FEI, Eindhoven, The Netherlands) was used to observe the distribution and morphology of NA. The precise molecular composition of gold nanocluster NA was determined using MALDI-TOF MS in positive-ion linear mode (ABI MALDI-TOF system, Foster, CA, USA), with 2,5-dihydroxybenzoic acid (DHB) as the matrix.

### 2.4. Au Content Measurement by ICP-MS

A 10 μL volume of NA solution was taken in a conical flask, and 4 mL of aqua regia was added to the conical flask and kept overnight. After digestion, the samples were flushed to the last drop of acid at 160 °C and diluted to 10 mL with 1% HCl and 2% HNO_3_ (Sinopharm Chemical Reagent, Beijing, China). Gold standard curves were obtained by using dilute mixed acids to prepare a series of gold standards with known concentrations (Au content of 0.5, 1, 5, 10, 50, and 100 ppb). The concentration of gold clusters can be obtained by comparing the test results of diluted samples with the gold standard curve.

For the determination of gold concentration in cells, cells (GBM cells, splenocytes, and bone marrow-derived cells) were seeded in 24-well plates at a density of 5 × 10^4^ cells/well and were incubated with 50 μM NA/GA. Two hours later, the same number of cells were collected by counting and digested by aqua regia. The remaining steps were the same as above.

For Au content measurement of tissue samples, the weighed tissue was pre-digested overnight by soaking in H_2_O_2_ and HNO_3_ (1:3). The tissue was driven to the last drop and soaked in aqua regia for at least 4 h. The remaining steps were same as above.

### 2.5. The Nar Peptide Blocking Experiment

GL261 cells (2 × 10^5^) were seeded in a 12-well plate and cultured for 24 h. The cells were then fixed with 4% paraformaldehyde (BL539A, Biosharp, Hefei, China) for 20 min. After washing the cells with PBS, the cells were incubated with 5 mM Nar peptide for 1 h. An additional 500 µL of 50 μM NA was incubated with the cells for 1 h at room temperature in the dark. Then, the cells were washed with PBS, and the Au content was determined using ICP-MS (PerkinElmer, Waltham, MA, USA).

### 2.6. Cell Viability Assay

Cells were seeded in sterile 96-well plates at a density of 1 × 10^4^ cells per well in a 100 μL volume of suspension. On the next day, cells were treated with RSL3 (HY-100218A, MCE, Shanghai, China) or Erastin (HY-15763, MCE) with or without clusters, or ferrostatin-1 (HY-100579, MCE), or zinc protoporphyrin (HY-101193, MCE) for 24 h. At the end of treatment, the medium and CCK-8 (C0038, Beyotime, Shanghai, China) solution were prepared into a mixture (10:1), and 100 μL of mixture was added to each well and incubated in the dark for 1 h. The cells were then detected on a microplate reader (OD 450, Molecular Devices, Sunnyvale, CA, USA). The cell viability of the cells in each experimental group was calculated by Graphpad prism (Version 9.0).

### 2.7. Lipid Peroxidation Detection

Cells were treated for 12 h and then incubated with 10 μM of C11-BODIPY 581/591 (HY-D1301, MCE) for 30 min. Subsequently, the cells were washed twice with PBS, and confocal fluorescence microscopy (Nikon Ti-2 Imaging Systems, Tokyo, Japan) was used to detect the relevant fluorescence signals. ImageJ was used for the quantitative analysis of the images.

### 2.8. Cellular Ferrous Iron Detection

GL261 cells were seeded in confocal dishes and co-treated with 2 μM RSL3 and 100 μM GA/NA for 24 h. The cells were washed 3 times with a serum-free medium. FerroOrange (F374, Dojindo Laboratories, Mashiki, Japan) working solution with a concentration of 1 μM was added and incubated at 37 °C in a 5% CO_2_ incubator. The cells were observed under fluorescence microscopy, and the fluorescence intensity was analyzed by ImageJ.

### 2.9. RNA Extraction and Real-Time PCR

After six hours of treatment, cells were removed, and RNAeasy^TM^ Animal mRNA Isolation Kit (R0027, Beyotime) was used to extract RNA from the cells. Thermofisher’s NanoDrop (Waltham, MA, USA) was used to measure the concentration of RNA. PCR was carried out using a LightCycler^®^96 real-time PCR system (Roche, Basel, Switzerland) using PerfectStartTM Green qPCR SuperMix (TransGen Biotech, Beijing, China). cDNA was produced using TransScript^®^ All-in-One FirstStrand cDNA Synthesis Super Mix for qPCR (TransGen Biotech). The 2^−ΔΔCt^ technique was used to examine relative quantification. Table 1 lists the primers that were utilized.

### 2.10. Western Blotting

RIPA lysis buffer was used to extract the total protein. A BCA protein assay kit was used to determine the concentrations of each protein. To denature the protein, SDS-PAGE loading buffer (5×) was added to the isolated protein sample and heated to 100 °C for 6 min. After that, the samples were placed onto an electrophoresis-ready 10% Bis-Tris acrylamide gel. The proteins on the gel were moved to the PVDF membrane after around two hours. After that, the PVDF membrane was submerged for a full hour in a blocking solution. Primary antibodies to α7nAChR (K002857P, Solarbio, Beijing, China) were added, and the membranes were shaken all night at 4 °C. On the next day, after another hour of secondary antibody (A0208, Beyotime) incubation, SupersignalTM Western Blot Enhancer (46640, ThermoFisher) was added, and a gel imaging equipment (Tanon, Shanghai, China) was used to capture the chemiluminescence. Beta-Actin (bs-0061R, Bioss Antibodies, Beijing, China) was used as housekeeping protein.

### 2.11. 3D Tumor Spheroid Model

A total of 1000 GBM cells were used to make a 100 µL cell suspension containing 1.2% methylcellulose, which was subsequently added to a 96 U-shaped well plate (MS-9096UZ, Sumitomo, Tokyo, Japan) and centrifuged at 1200 rpm for 3 min. Transferring orifice was carefully placed in 5% CO_2_ and at 37 °C in an incubator culture, and cells gradually formed spheres within 24–48 h. Tumor spheres were transferred according to the experiment on 96-well plate for subsequent experiments.

The well-grown 3D spheroids were treated with RSL3 (2 μM) to induce ferroptosis in addition to 100 μM GA/NA. After 24 h, the 3D tumor spheres were stained with live/dead double staining using Calcein-AM (C2012, Beyotime) and PI (556463, BD Biosciences, Franklin Lakes, NJ, USA) for 30 min, and then, confocal imaging was used for statistical analysis, and 10 sections were obtained for each sphere by z-axis scanning. For each section, green and red cells were counted separately using the monochrome mode. Death index is used to describe mortality and to calculate the average data to describe the total mortality of a sphere:

$$Death\;Index\;for\;each\;scanned\;layer=\frac{Red\;pixel\;area}{\left(Green+Red\right)\;pixel\;area}\times 100\%$$

### 2.12. Animal Experiments

Six-week-old male C57BL/6J mice, purchased from Beijing HFK Bioscience Co., Ltd., were kept in standard laboratory conditions. The protocols for all animal studies were carried out in compliance with the National Law on the Use of Experimental Animals as well as the Animal Care and Requirements of Beijing University of Technology’s Ethics Committee.

A total of 5 × 10^6^ GL261 cells were subcutaneously injected into C57BL/6J mice. Once the tumors were evident, treatment began. At the beginning of therapy, mice were randomly assigned to four groups and given intraperitoneal injections of RSL3 (2.5 mg/kg), GA (10 mg/kg), and NA (10 mg/kg) every other day. The size of the tumor was measured with a caliper every day. The formula for calculating the tumor volume was (length × width^2^) × 0.5. After 13 days, the mice were euthanized, and primary organs, blood, and tumor tissue were taken. Tumor tissues were prepared for ICP-MS, immune cell flow analysis, Fe content measurement, and immunofluorescence staining. Main organs were extracted and stained with HE. Whole blood and blood serum were tested for routine blood test and different biochemical indicators, respectively.

### 2.13. Immunofluorescence Staining of HO-1

Paraffin-embedded tumor slices were first incubated with an anti-HO-1 antibody (dilution 1:100, bs-23397R, Bioss Antibodies) at 4 °C overnight. Following this, the sections were stained with a Goat anti-rabbit IgG H&L (Alexa Fluor 594) secondary antibody (dilution 1:500, ab150080, abcam, Branford, CT, USA) in the dark for 30 min. Next, the sections were counterstained with DAPI (S2110, Solarbio) for 10 min. After mounting, the samples were observed and scanned under a microscope. The area of individual molecules was measured using the ImageJ software (Version 1.54j).

### 2.14. Fe Content Measurement

The iron content in the samples was measured following the manufacturer’s instructions. Briefly, tumor tissues weighing more than 0.1 g were homogenized, and the total iron content was determined using a tissue iron content assay kit (BC4355, Solarbio).

### 2.15. Flow Cytometry

The tumors were cut with scissors, then digested with collagenase IV (17104019, Gibco, Waltham, MA, USA) and DNase I (10104159001, Roche) at the concentration of 1 mg/mL and 0.2 mg/mL, respectively, and incubated at 37 °C for 30 min for full digestion. Cells were then subjected to a 70 μm filter and then centrifuged at 1800 rpm at 4 °C for 4 min. After centrifugation, 2 × 10^6^ cells were counted, and 1 μL zombie NIR antibody (423105, Biolegend, San Diego, CA, USA) was added for live and dead cell staining. The cells were centrifuged at room temperature in the dark for 20 min and centrifuged at 1800 rpm for 4 min. Then, cells were stained by BV510-CD45 (103137, Biolegend), PerCP/Cy5.5-CD3 (100217, Biolegend), FITC-CD4 (100406, Biolegend), APC-CD8a (100711, Biolegend), and PE-CD62L (161203, Biolegend) in the dark for another 20 min. Then, flow cytometry was conducted (Cytek Northern Lights-CLC 3000, Fremont, CA, USA). The data were analyzed using the FlowJo software (Version 10.0).

### 2.16. Blood Biochemical Indicators and Blood Routine Indicators

At the humanitarian end of the experiment, blood samples were collected from the mice for serum separation and sent to Beijing soonbio Technology Co., Ltd. (Beijing, China) for the biochemical detection of cardiac-related indicators. Whole blood samples were sent to Beijing servicebio Co., Ltd. (Beijing, China) for routine blood test.

### 2.17. Statistics

All statistical results in this study were expressed as mean ± SEM. Differences were considered to be statistically significant when the *p* value was <0.05. Data were analyzed by Students’ *t*-test.

## 3. Results and Discussion

### 3.1. Synthesis and Characterization of GBM-Targeting Gold Cluster NA

**Figure 1:**
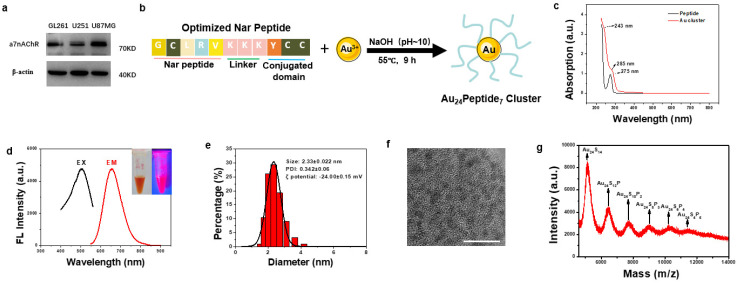
Synthesis and Characterization of NA. (**a**) Western blot analysis of α7nAChR expression in GBM cell lines. (**b**) Schematic diagram of the synthetic route of NA. (**c**) The UV-Vis spectrum analysis of NA and Nar peptide. (**d**) Optimal fluorescence excitation (black line, λ = 504 nm) and emission (red line, λ = 664 nm) spectra of NA. The inset are images of NA under visible light (left) and 365 nm UV light (right), respectively. (**e**) Dynamic light scattering analysis of NA. (**f**) HRTEM image of the synthesized NA, scale bar = 10 nm. (**g**) The molecular composition of NA was analyzed by MALDI-TOF MS.

To design a GBM-targeting gold cluster, we assessed the literature and found that α7 nicotinic acetylcholine receptor (α7nAChR) has been reported to express on tumors from the central nervous system [23,24]. We examined the expression of α7nAChR on several GBM cell lines, including mice GL261 and human U251 and U87MG, and found that α7nAChR was highly expressed on these GBM cells, confirming α7nAChR as a viable target in GBM (Figure 1a). Therefore, we adopted and optimized the reported α7nAChR-targeting peptide (Nar) as a ligand to functionalize gold clusters to facilitate their targeting of GBM cells. The sequence of the optimized Nar peptide was Gly-Cys-Leu-Arg-Val-Lys-Lys-Lys-Tyr-Cys-Cys. Among them, Gly-Cys-Leu-Arg-Val was the reported GBM-targeting peptide, while Lys-Lys-Lys increased the hydrophilicity. Cys-Cys was used to anchor gold atoms, which were reduced by Tyr. The synthesis of GBM-targeting peptide-modified gold clusters, designated as NA, was straightforward (Figure 1b). UV-Vis spectroscopy revealed an absorption peak at approximately 275 nm corresponding to the Nar peptide, with a new peak at 285 nm emerging upon gold cluster formation, indicative of the phenolic hydroxyl group reaction and successful cluster synthesis (Figure 1c). The aqueous NA solution was dark yellow under normal visible light and had obvious pink fluorescence under the ultraviolet light. According to the fluorescence spectrum detection, the best fluorescence emission position of NA was 664 nm (red curve) under the optimal excitation wavelength of 504 nm (black curve) (Figure 1d). Dynamic light scattering (DLS) analysis showed that the average hydrated diameter of NA was 2.33 ± 0.022 nm, with PDI of 0.342 ± 0.06 (Figure 1e). The zeta potential of NA was −24 mV (Figure 1e). High-resolution transmission electron microscopy (HRTEM) revealed uniform distribution and particle size of the NA (Figure 1f). Matrix-assisted laser desorption/ionization time-of-flight mass spectrometry (MALDI-TOF MS) is used to analyze the precise molecular composition of NA. The highest peak intensity belonged to Au_24_S_14_, considering the spacing between adjacent peaks matched the molecular weight of a single Nar peptide, and each peptide has two cysteine residues, and the molecular formula for the gold cluster is Au_24_Peptide_7_ (Figure 1g).

### 3.2. NA Exhibits Specific Targeting Capability towards GBM In Vitro and In Vivo

**Figure 2:**
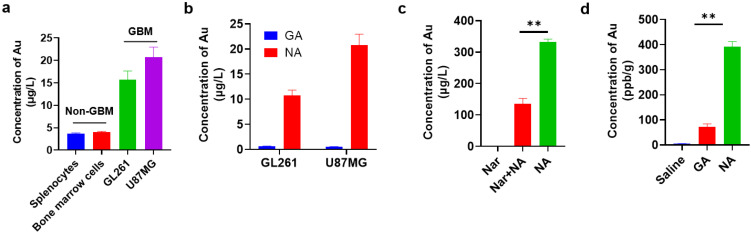
NA can effectively target GBM cells and GBM tumors in vitro and in vivo. (**a**) ICP-MS quantification of NA uptake by 1 × 10^5^ indicated non-GBM and GBM cells. Non-GBM, not GBM cells; GBM, GBM cell lines. (**b**) ICP-MS quantification of GA/NA uptake by 1 × 10^5^ indicated GBM cells. (**c**) Content of gold was measured by ICP-MS in 1 × 10^5^ GL261 cells after being treated with NA (50 μM) for 2 h with or without Nar peptide (5 mM) pre-treatment. (**d**) Mice were inoculated with GL261 cells for 7 days. Six hours after intraperitoneal injection of clusters (10 mg/kg) into tumor-bearing mice, the content of Au in tumors was detected by ICP-MS. Data are presented as the mean ± SEM (*n* = 3). ** *p* < 0.01.

To identify the targeting capability of NA towards GBM, we first compared the uptake of clusters by GBM cell lines and non-GBM cells by inductively coupled plasma mass spectrometry (ICP-MS). We found that the NA uptake of GBM cells was nearly 20-fold higher compared to that of non-GBM cells under identical conditions (Figure 2a). To further confirm this, we also synthesized a non-targeting gold cluster by adopting tripeptide γ-GSH as the conjugating peptide, namely, GA. Compared to NA, GA possessed red fluorescence, and its average hydrated diameter was 1.70 ± 0.013 nm (Figure S1). Similar analysis showed that the targeting efficacy of NA towards GBM cells was significantly reinforced in comparison with GA (Figure 2b). To validate that the uptake of NA was mediated through α7nAChR recognition, cells pre-treated with NA were subsequently incubated with high concentrations of Nar peptide to competitively bind α7nAChR receptors on the cell membrane. This pre-incubation significantly reduced NA uptake in GBM cells, as indicated by ICP-MS (Figure 2c). Moreover, to compare the in vivo targeting efficiency of NA and GA, both clusters were administered at equivalent doses to tumor-bearing mice, and tumor tissues were collected after six hours. Consistently, the gold content in tumors of NA-treated mice was significantly higher than that in mice treated with GA (Figure 2d). Collectively, these findings demonstrate that NA can effectively target GBM.

### 3.3. NA Significantly Sensitized GBM Cells to Ferroptosis

**Figure 3:**
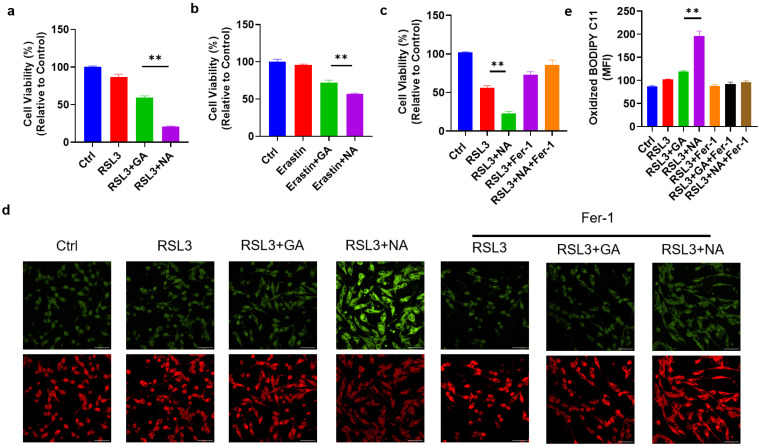
NA enhanced ferroptosis levels in GBM cells. (**a**) GL261 cells were treated with RSL3 (2 μM) with and without GA/NA (100 μM) for 24 h, and CCK-8 was used to detect cell viability. (**b**) GL261 cells were treated with Erastin (5 μM) with and without GA/NA (100 μM) for 24 h, and the cell viability was tested by CCK-8. (**c**) GL261 cells were treated similarly as in (**a**), with the addition of Fer-1 (20 μM) treatment. (**d**,**e**) GL261 cells were treated with RSL3 (2 μM) with and without GA/NA (100 μM) for 24 h, followed by staining with BODIPY C11. Cells were visualized using laser confocal microscopy. Representative images (**d**) and statistics (**e**) were shown. Scale bar = 25 μm. Data are presented as the mean ± SEM (*n* = 3). ** *p* < 0.01.

To assess the cytotoxic effects of the targeted gold cluster NA on GBM cells, we first treated GMB cell lines with NA alone and found that NA did not impact cell viability (Figure S2). Given the use of ferroptosis-related drugs in GBM treatment, we hypothesized that NA could sensitize GBM cells to ferroptosis. Thus, RSL3, a common ferroptosis inducer, was applied to induce ferroptosis in GL261 cells. As anticipated, RSL3 reduced the viability of GL261 cells. Moreover, treatment with either GA or NA at equivalent doses increased the sensitivity of GL261 cells to ferroptosis, with NA showing a much more pronounced effect (Figure 3a). This enhanced sensitization to ferroptosis in GBM cells by NA is attributable to the greater accumulation in GBM cells compared to GA. A similar outcome was observed with another classic ferroptosis inducer, Erastin (Figure 3b). We further employed ferroptosis inhibitor Fer-1 and found that Fer-1 completely reversed the effect of NA, indicating that NA-induced cell death occurs solely due to ferroptosis (Figure 3c). To further substantiate that NA promotes ferroptosis in GBM cells, BODIPY C11, a lipid peroxidation probe, was used to stain treated cells. We found the co-treatment with NA and RSL3 maximized lipid peroxidation levels. Conversely, Fer-1 reversed lipid peroxidation and inhibited ferroptosis (Figure 3d,e).

### 3.4. NA Activates the Non-Canonical Signaling Pathway of Ferroptosis

**Figure 4:**
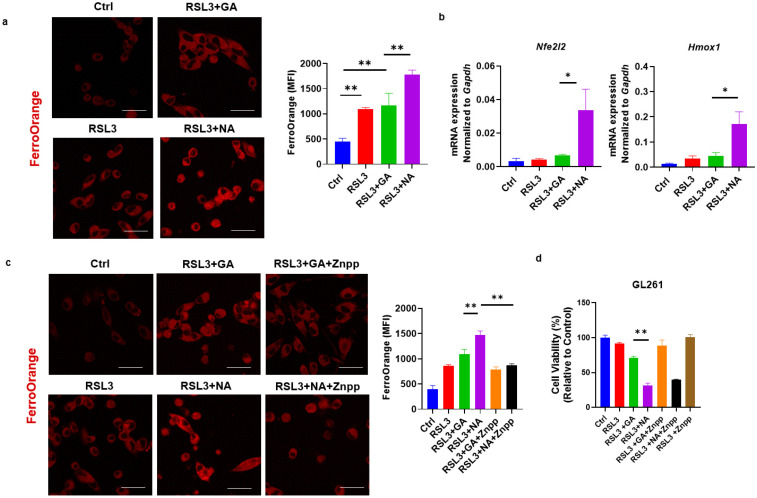
NA specifically activated the non-canonical ferroptosis in GBM cells. (**a**) Confocal images of GL261 cells treated with indicated treatments following FerroOrange staining, and the statistical analysis are shown. Scale bars = 10 μm. (**b**) qPCR detection of transcription levels of Nrf2/HO-1 under the treatment of NA. (**c**) Confocal images of FerroOrange-stained GL261 cells under the action of RSL3, GA, NA, and HO-1 inhibitor Znpp (5 μM). Scale bars = 10 μm. (**d**) The effect of Znpp on the cell viability of GL261 cells is shown. Data are presented as the mean ± SEM (*n* = 3).* *p* < 0.05, ** *p* < 0.01.

Iron metabolism is an important aspect of the activation and regulation of ferroptosis. In the non-canonical pathway of ferroptosis, the excessive activation of HO-1 increases the levels of ferrous iron (Fe^2+^) within the labile iron pool, catalyzing the formation of free radicals and inducing lipid peroxidation via the Fenton reaction [25,26]. Our previous research demonstrated that gold clusters enhance HO-1 expression in cells [7]. This led us to investigate whether the enhanced efficacy of ferroptosis inducers by gold clusters is associated with non-canonical ferroptosis pathways. Using FerroOrange staining, we observed that co-treatment with NA and RSL3 significantly increased Fe^2+^ concentration in GL261 cells (Figure 4a). To further elucidate the mechanism of intracellular Fe^2+^ increase, we examined the expression levels of Nrf2-HO-1, the key component of the non-canonical ferroptosis pathway, following treatment with gold clusters. Our results showed that NA, compared to GA, significantly upregulated the transcriptional expression of these genes (Figure 4b). Znpp, the specific small-molecule inhibitor of HO-1, reduced the accumulation of Fe^2+^ in cells and restored cell viability, further confirming the role of the HO-1 signaling pathway in NA-induced cell death (Figure 4c,d). These findings suggest that NA exerts its effects specifically through the non-canonical ferroptosis pathway. By activating the Nrf2-HO-1 signaling pathway, heme oxygenase degrades heme, leading to an increase in intracellular Fe^2+^ concentration, which subsequently promotes lipid peroxidation and ferroptosis.

### 3.5. NA Combined with a Ferroptosis Inducer Inhibits the Growth of GBM Spheroids

**Figure 5:**
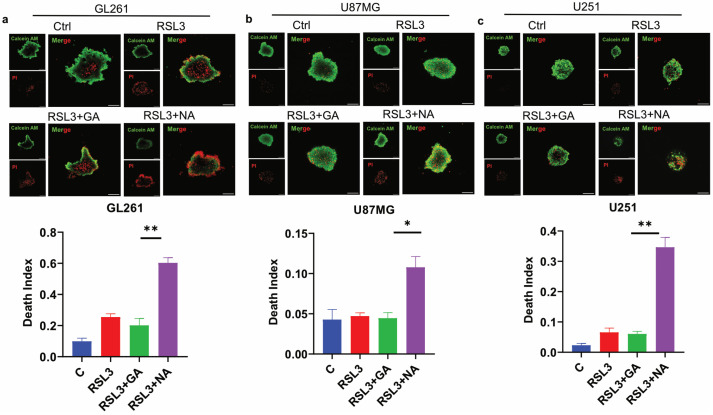
NA induced ferroptosis in GBM tumor 3D spheroids. (**a**–**c**) Calcein AM/PI double staining of 3 GBM cell lines, and tumor 3D spheroids were treated by stimulus (RSL: 3:5 μM; GA/NA: 100 μM), and the statistics of death index are shown. Scale bar = 100 μm. Data are presented as the mean ± SEM (*n* = 3). * *p* < 0.05, ** *p* < 0.01.

Three-dimensional (3D) tumor spheroid has emerged as a crucial in vitro model for cancer research, as it more accurately mimics the in vivo structure of solid tumors compared to traditional 2D cell cultures, thereby providing a better indication of anti-tumor efficacy [27,28]. To evaluate the effect of NA on ferroptosis in GBM tumor 3D spheroids, we conducted assays on spheroids derived from three GBM cell lines. Calcein AM and PI staining were employed to differentiate live cells from dead cells, and the death index was calculated as the ratio of the red area to the green area. Our findings demonstrated that the combination of NA and RSL3 significantly increased the proportion of dead cells in the tumor spheroids from all three GBM cell lines (Figure 5a–c). Similarly, NA combined with Erastin induced comparable effects in GL261 tumor spheroids (Figure S3). However, GA did not have a significant death-promoting effect in these three tumor spheres, which may be due to the fact that the efficiency of GA in these GBM cells is not as high as that of NA, and the difficulty of drug entry into cells is further increased in spheroids.

### 3.6. NA Significantly Enhances Tumor Ferroptosis and Inhibits Tumor Growth

**Figure 6:**
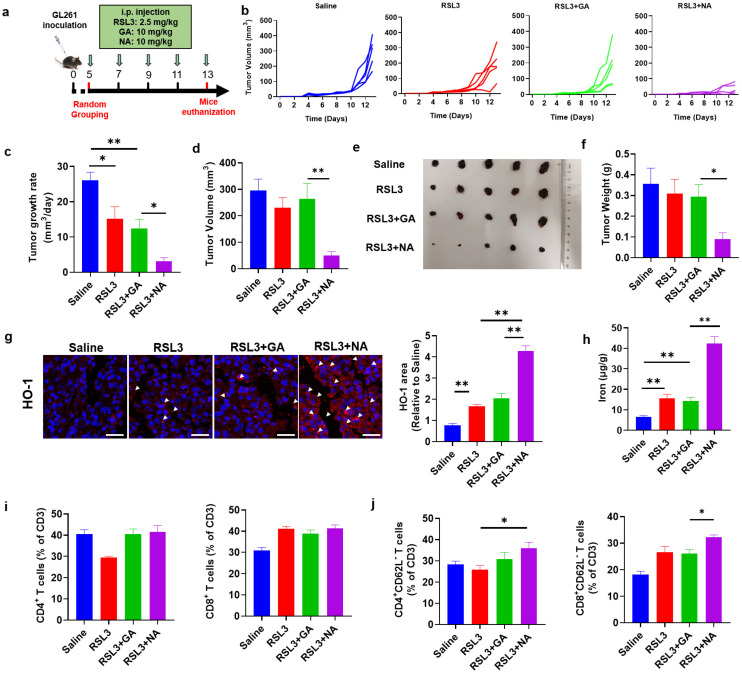
NA suppresses GBM in vivo. (**a**) Schematic diagram of the therapeutic tumor model. (**b**) Tumor growth curves plotted daily according to tumor volume. (**c**) The tumor growth rate is shown. (**d**) Tumor volume recorded at the end of treatment. (**e**) Photograph of the tumor dissected at the end of treatment. (**f**) The tumor weight is shown. (**g**) The images of immunofluorescence staining of HO-1 in tumor tissues. Scale bar = 100 μm. (**h**) The content of ferrous ions in tumor tissues. (**i**) The percentage of CD4- and CD8-positive immune cells in tumors after treatment. (**j**) The percentage of indicated immune cell subtypes in tumors after treatment. Data are presented as the mean ± SEM (*n* = 3). * *p* < 0.05, ** *p* < 0.01.

We have demonstrated that NA promotes ferroptosis in GBM cell lines in vitro. Given its strong tumor-targeting capability, we hypothesized that NA, in combination with ferroptosis inducers, would also exhibit potent anti-tumor effects in vivo. To test this, we established a GBM model by injecting GL261 cells subcutaneously (Figure 6a). While RSL3 combined with GA did not significantly inhibit the tumor volume growth, the NA-treated group showed a marked reduction in tumor growth (Figure 6b–d). Similarly, in addition to volumetric changes, NA combined with low doses of RSL3 was also able to significantly suppress tumor weight (Figure 6e,f).

NA synergizes with ferroptosis inducers to promote tumor cell ferroptosis in vitro. To confirm whether NA also inhibits tumor growth by promoting ferroptosis in vivo, we detected HO-1 and Fe^2+^ levels in tumor tissues. The combination of NA and RSL3 significantly increased the expression of HO-1 and the concentration of Fe^2+^ in tumors, suggesting that NA enhanced tumor ferroptosis by upregulating HO-1 expression (Figure 6g,h).

Tumor cell death was widely considered to activate anti-tumor immune responses. Especially, ferroptosis was also reported to exert a promoting role on anti-tumor immunity [29]. To investigate if the ferroptosis induced by NA can also change the intratumoral immune responses, we analyzed the tumor-infiltrated immune cells by flow cytometry. We found that, although NA did not change the percentage of CD4^+^ or CD8^+^ T cells (Figure 6i), the proportions of CD4^+^CD62L^−^ and CD8^+^CD62L^−^ T cells (tumoricidal effector T cells [30]) were both significantly elevated (Figure 6j). These results indicated that NA not only promoted tumor ferroptosis but also activated anti-tumor immunity, leading to significant anti-tumor effects.

### 3.7. NA Demonstrates Excellent Biosafety

**Figure 7:**
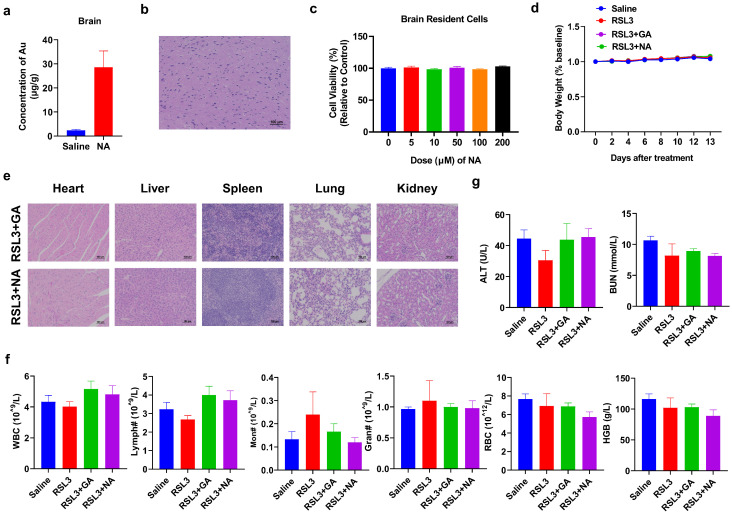
Biosafety analysis of NA. (**a**,**b**) Saline or NA (10 mg/kg) was i.p. injected, and the brain was isolated after 6 h and subjected to (**a**) ICP-MS or (**b**) HE staining, scale bar = 100 µm. (**c**) Brain cells were isolated and incubated with NA for 24 h, and then, the CCK-8 test was conducted. (**d**) Changes in body weight in each group during treatment. (**e**) HE staining results of major organs of tumor-bearing mice after treatment. Scale bar = 100 µm. (**f**) The blood test result of tumor-bearing mice by indicated treatments. WBC: white blood cells; Lymph: lymphocytes; Mon: monocytes; Gran: granulocytes; RBC: red blood cells; and HGB: hemoglobin. (**g**) Blood biochemical analysis associated with cardiotoxicity was tested.

While the combination therapy strategy has shown promising anti-tumor effects, the biosafety of nanomaterials is equally critical. Since GBM originally occurred in the brain, we tested if NA can across the BBB to penetrate into the brain area and cause any damage to normal brain cells. To test this, we performed ICP-MS on the brain of NA-treated mice and found that the concentration of NA was significantly higher than that in the control group (Figure 7a). Furthermore, both the brain section from NA-treated mice and the brain resident cells treated with NA in vitro showed no detrimental effect (Figure 7b,c). We also monitored the body weight of tumor-bearing mice post-treatment and found no significant weight loss in any group throughout the treatment cycle (Figure 7d). The histological sections of the main organs (the heart, liver, spleen, lung, and kidney) were stained with HE, and no obvious damage was observed in the organs and tissues (Figure 7e). The number and percentage of blood cells, as well as the level of critical proteins, were all within the normal range (Figure 7f and Figure S4). More importantly, ferroptosis inducers have been reported to cause significant cardiotoxicity [31]. Therefore, we performed the blood biochemical detection of cardiac-related indicators, including alanine aminotransferase (ALT) and blood urea nitrogen (BUN), and found that the indices were also within the normal range (Figure 7g). Overall, the hematological indices and structural integrity of major organs suggest that NA is highly biocompatible and biologically safe.

## 4. Conclusions

In summary, we have developed a gold cluster NA that can efficiently target glioblastoma. Due to the characteristics of NA, it possesses excellent biosafety and can penetrate the BBB. Meanwhile, NA can significantly increase the ferroptosis level of glioblastoma by activating the non-canonical ferroptosis signaling pathway and subsequently promote anti-tumor immune responses, thus exerting a profound anti-tumor effect. This study may provide critical information and potential new strategies for the treatment of glioblastoma.

## Figures and Tables

**Table 1:** The primer sequences used in the study.

Gene	Forward Primer (5′→3′)	Reverse Primer (5′→3′)
GAPDH	AGG TCG GTG TGA ACG GAT TTG	GGG GTC GTT GAT GGC AAC A
Hmox-1	AGC CCC ACC AAG TTC AAA CA	CAT CAC CTG CAG CTC CTC AA
Nfe2l2	CTT TAG TCA GCG ACA GAA GGA C	AGG CAT CTT GTT TGG GAA TGT G

## Data Availability

Data will be made available on request.

## Supplementary Materials

The following supporting information can be downloaded at: https://www.mdpi.com/article/10.3390/pharmaceutics16091205/s1, Figure S1: Characterization of GA; Figure S2: Viability of NA-treated GBM cell lines; Figure S3: Staining of GBM tumor 3D spheroids treated with NA combined with Erastin; Figure S4: The blood test result of tumor bearing mice treated with ferroptosis inducer and cluster.

## References

[1] Schaff L.R., Mellinghoff I.K. (2023). Glioblastoma and Other Primary Brain Malignancies in Adults: A Review. JAMA.

[2] Lah T.T., Novak M., Breznik B. (2020). Brain malignancies: Glioblastoma and brain metastases. Semin. Cancer Biol..

[3] Czarnywojtek A., Borowska M., Dyrka K., Van Gool S., Sawicka-Gutaj N., Moskal J., Kościński J., Graczyk P., Hałas T., Lewandowska A.M. (2023). Glioblastoma Multiforme: The Latest Diagnostics and Treatment Techniques. Pharmacology.

[4] Zhu R., Zhang F., Peng Y., Xie T., Wang Y., Lan Y. (2022). Current Progress in Cancer Treatment Using Nanomaterials. Front. Oncol..

[5] Wei D., Zhang N., Qu S., Wang H., Li J. (2023). Advances in nanotechnology for the treatment of GBM. Front. Neurosci..

[6] van de Looij S.M., Hebels E.R., Viola M., Hembury M., Oliveira S., Vermonden T. (2022). Gold Nanoclusters: Imaging, Therapy, and Theranostic Roles in Biomedical Applications. Bioconjug Chem..

[7] Lu C., Xue L., Luo K., Liu Y., Lai J., Yao X., Xue Y., Huo W., Meng C., Xia D. (2023). Colon-Accumulated Gold Nanoclusters Alleviate Intestinal Inflammation and Prevent Secondary Colorectal Carcinogenesis via Nrf2-Dependent Macrophage Reprogramming. ACS Nano.

[8] Pang Z., Yan W., Yang J., Li Q., Guo Y., Zhou D., Jiang X. (2022). Multifunctional Gold Nanoclusters for Effective Targeting, Near-Infrared Fluorescence Imaging, Diagnosis, and Treatment of Cancer Lymphatic Metastasis. ACS Nano.

[9] Xiao F., Chen Y., Qi J., Yao Q., Xie J., Jiang X. (2023). Multi-Targeted Peptide-Modified Gold Nanoclusters for Treating Solid Tumors in the Liver. Adv. Mater..

[10] Gao X., Cao K., Yang J., Liu L., Gao L. (2024). Recent advances in nanotechnology for programmed death ligand 1-targeted cancer theranostics. J. Mater. Chem. B.

[11] Li H., Li J., Wang M., Feng W., Gao F., Han Y., Shi Y., Du Z., Yuan Q., Cao P. (2023). Clusterbody Enables Flow Sorting-Assisted Single-Cell Mass Spectrometry Analysis for Identifying Reversal Agent of Chemoresistance. Anal. Chem..

[12] Lu C., Meng C., Li Y., Yuan J., Ren X., Gao L., Su D., Cao K., Cui M., Yuan Q. (2024). A probe for NIR-II imaging and multimodal analysis of early Alzheimer’s disease by targeting CTGF. Nat. Commun..

[13] Hu J., Gao G., He M., Yin Q., Gao X., Xu H., Sun T. (2020). Optimal route of gold nanoclusters administration in mice targeting Parkinson’s disease. Nanomedicine.

[14] Mahapatra A., Sarkar S., Biswas S.C., Chattopadhyay K. (2020). Modulation of α-Synuclein Fibrillation by Ultrasmall and Biocompatible Gold Nanoclusters. ACS Chem. Neurosci..

[15] Nair L.V., Nair R.V., Shenoy S.J., Thekkuveettil A., Jayasree R.S. (2017). Blood brain barrier permeable gold nanocluster for targeted brain imaging and therapy: An in vitro and in vivo study. J. Mater. Chem. B.

[16] Jiang X., Stockwell B.R., Conrad M. (2021). Ferroptosis: Mechanisms, biology and role in disease. Nat. Rev. Mol. Cell Biol..

[17] Cao K., Tait S.W.G. (2018). Apoptosis and Cancer: Force Awakens, Phantom Menace, or Both?. Int. Rev. Cell Mol. Biol..

[18] Fang X., Ardehali H., Min J., Wang F. (2023). The molecular and metabolic landscape of iron and ferroptosis in cardiovascular disease. Nat. Rev. Cardiol..

[19] Yao Y., Lu C., Gao L., Cao K., Yuan H., Zhang X., Gao X., Yuan Q. (2021). Gold Cluster Capped with a BCL-2 Antagonistic Peptide Exerts Synergistic Antitumor Activity in Chronic Lymphocytic Leukemia Cells. ACS Appl. Mater. Interfaces.

[20] Yu Y., Yan Y., Niu F., Wang Y., Chen X., Su G., Liu Y., Zhao X., Qian L., Liu P. (2021). Ferroptosis: A cell death connecting oxidative stress, inflammation and cardiovascular diseases. Cell Death Discov..

[21] Tan Y., Huang D., Luo C., Tang J., Kwok R.T.K., Lam J.W.Y., Sun J., Liu J., Tang B.Z. (2023). In Vivo Aggregation of Clearable Bimetallic Nanoparticles with Interlocked Surface Motifs for Cancer Therapeutics Amplification. Nano Lett..

[22] Qu X., Yin F., Pei M., Chen Q., Zhang Y., Lu S., Zhang X., Liu Z., Li X., Chen H. (2023). Modulation of Intratumoral Fusobacterium nucleatum to Enhance Sonodynamic Therapy for Colorectal Cancer with Reduced Phototoxic Skin Injury. ACS Nano.

[23] Nagele R.G., D’Andrea M.R., Anderson W.J., Wang H.Y. (2002). Intracellular accumulation of beta-amyloid(1–42) in neurons is facilitated by the alpha 7 nicotinic acetylcholine receptor in Alzheimer’s disease. Neuroscience.

[24] Kumar P., Wu H., McBride J.L., Jung K.E., Kim M.H., Davidson B.L., Lee S.K., Shankar P., Manjunath N. (2007). Transvascular delivery of small interfering RNA to the central nervous system. Nature.

[25] Stockwell B.R. (2022). Ferroptosis turns 10: Emerging mechanisms, physiological functions, and therapeutic applications. Cell.

[26] Stockwell B.R., Friedmann Angeli J.P., Bayir H., Bush A.I., Conrad M., Dixon S.J., Fulda S., Gascón S., Hatzios S.K., Kagan V.E. (2017). Ferroptosis: A Regulated Cell Death Nexus Linking Metabolism, Redox Biology, and Disease. Cell.

[27] Nunes A.S., Barros A.S., Costa E.C., Moreira A.F., Correia I.J. (2019). 3D tumor spheroids as in vitro models to mimic in vivo human solid tumors resistance to therapeutic drugs. Biotechnol. Bioeng..

[28] Zhao L., Xiu J., Liu Y., Zhang T., Pan W., Zheng X., Zhang X. (2019). A 3D Printed Hanging Drop Dripper for Tumor Spheroids Analysis Without Recovery. Sci. Rep..

[29] Zheng Y., Sun L., Guo J., Ma J. (2023). The crosstalk between ferroptosis and anti-tumor immunity in the tumor microenvironment: Molecular mechanisms and therapeutic controversy. Cancer Commun.

[30] Künzli M., Masopust D. (2023). CD4(+) T cell memory. Nat. Immunol..

[31] Wang K., Chen X.Z., Wang Y.H., Cheng X.L., Zhao Y., Zhou L.Y., Wang K. (2022). Emerging roles of ferroptosis in cardiovascular diseases. Cell Death Discov..

