# Supplementary material for: Induction of Non-Canonical Ferroptosis by Targeting Clusters Suppresses Glioblastoma
Source: Pharmaceutics. 2024 Sep 13;16(9):1205. doi: 10.3390/pharmaceutics16091205 (PMC11434859; doi:10.3390/pharmaceutics16091205)
Supplement: Supplementary file 1 [file pharmaceutics-16-01205-s001.zip › pharmaceutics-3195511-supplementary.pdf]

Supplementary Materials

Induction of Non-Canonical Ferroptosis by Targeting Clusters Suppresses Glioblastoma

Kai Cao †, Liyuan Xue †, Kaidi Luo, Wendi Huo, Panpan Ruan, Dongfang Xia, Xiuxiu Yao, Wencong Zhao, Liang Gao * and Xueyun Gao *

Center of Excellence for Environmental Safety and Biological Effects, Department of Chemistry, College of Chemistry and Life Science, Beijing University of Technology, Beijing 100124, China

* Correspondence: gaoliang@bjut.edu.cn (L.G.); gaoxy@ihep.ac.cn (X.G.)

† These authors contributed equally to this work.

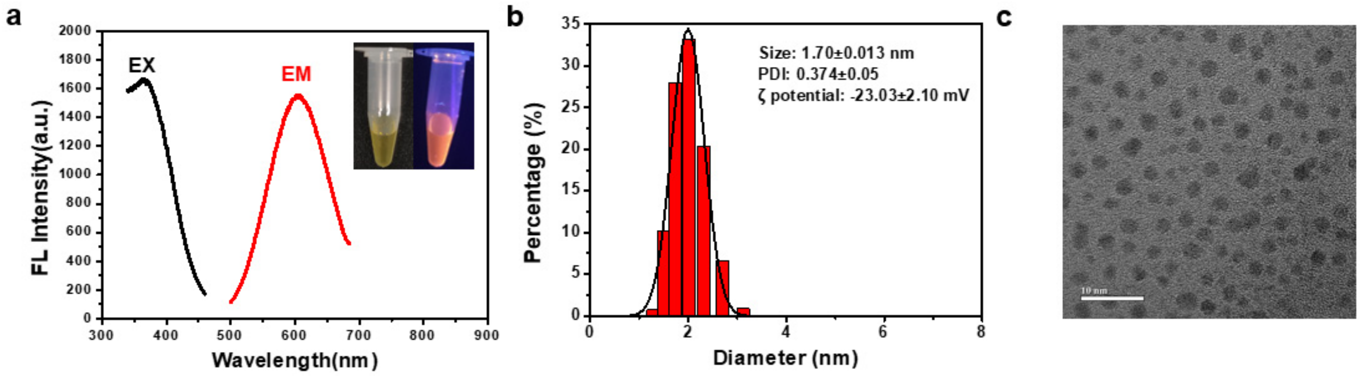

Figure S1. (**a**) Optimal fluorescence excitation (black line) and emission (red line) spectra of GA. The inset are images of GA under visible light (left) and 365 nm UV light (right), respectively. (**b**) Dynamic light scattering and zeta petential analysis of GA. (**c**) HRTEM image of the synthesized GA, scale bar = 10 nm.

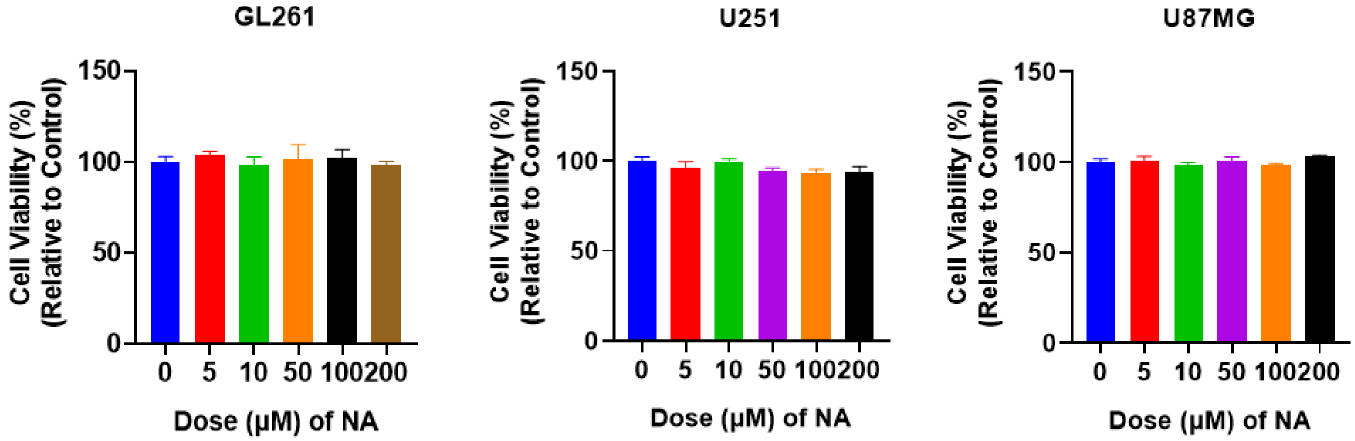

Figure S2. The viability of GL261, U251 and U87MG cells treated with different doses of NA.

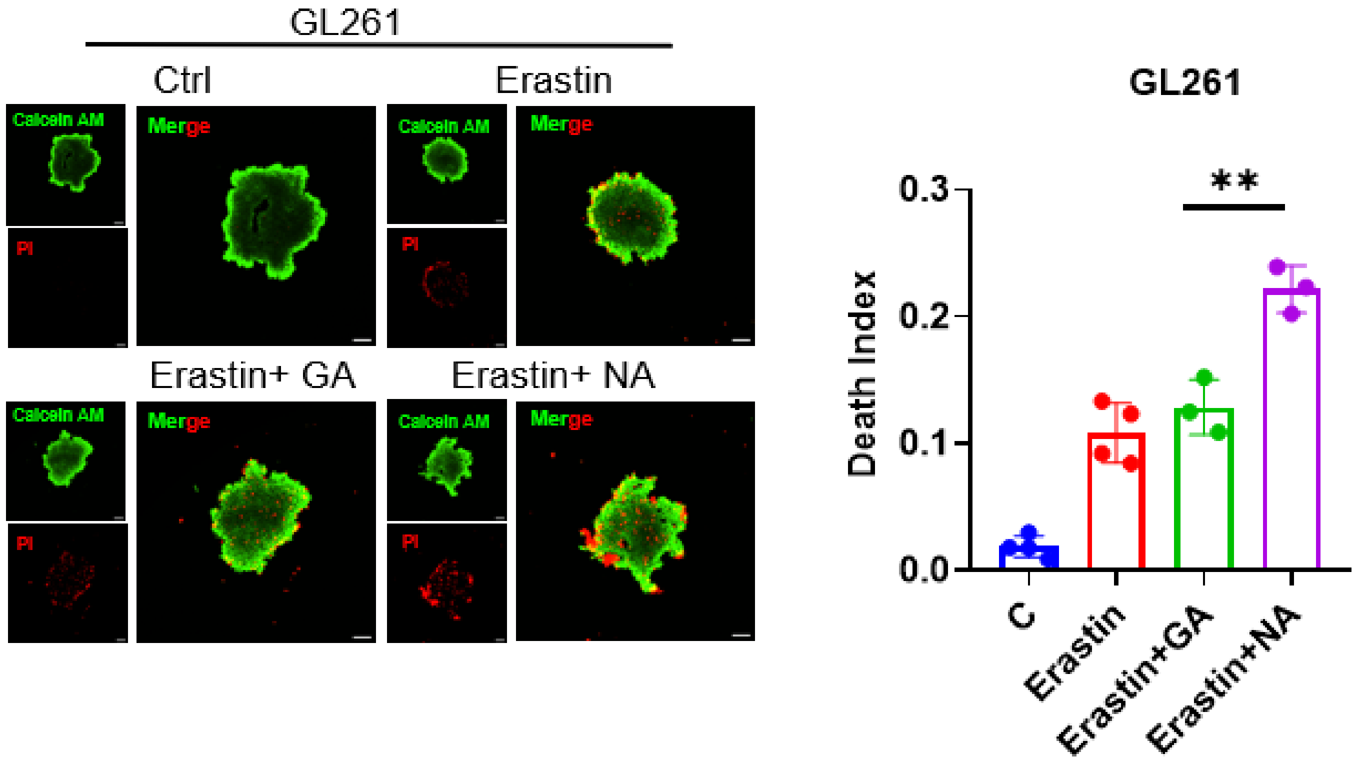

Figure S3. Calcin-AM /PI double staining of GL261 tumor 3D spheroids were treated by stimulus(Erastin: 10 μM, GA/NA: 100 μM) and the statistics of death index. Scale bar = 100 μm. ** $p < 0.01$.

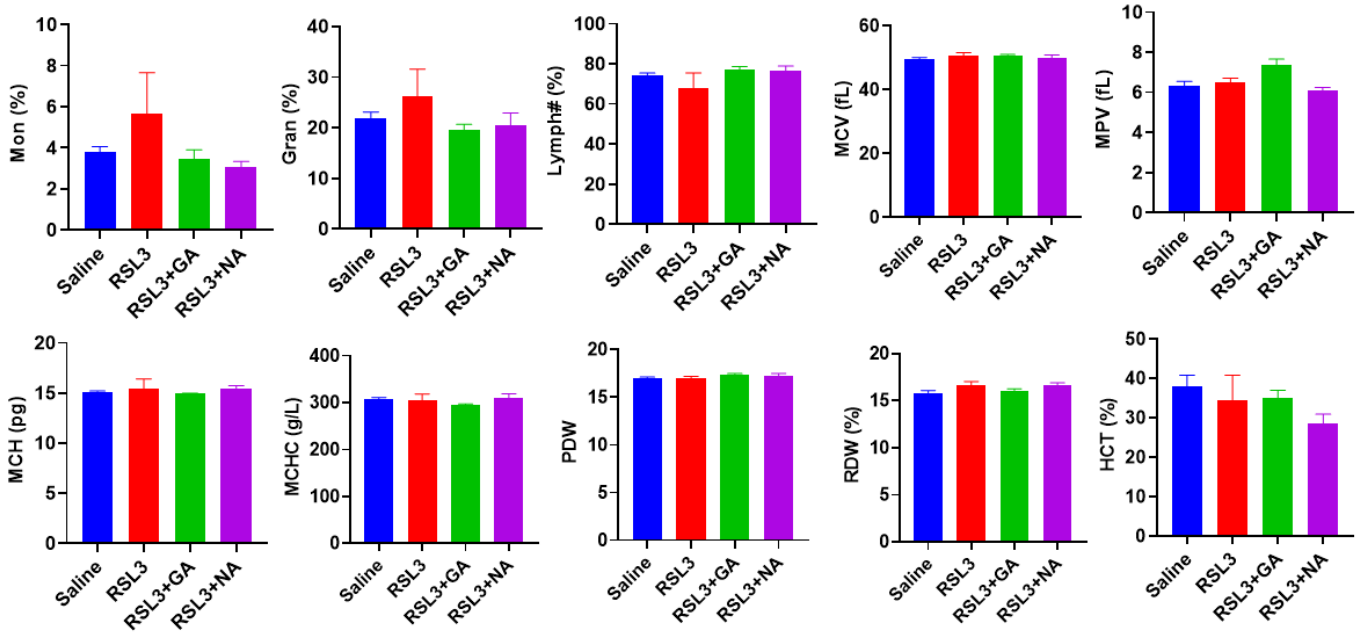

Figure S4. The blood test result of tumor-bearing mice by indicated treatments. MCV: mean corpuscular volume; MPV: mean platelet volume; MCH: mean corpuscular hemoglobin; MCHC: mean corpuscular hemoglobin concentration; PDW: platelet distribution width; RDW: red blood cell distribution width; HCT: hematocrit.
